# KaruBioNet: a network and discussion group for a better collaboration and structuring of bioinformatics in Guadeloupe (French West Indies)

**DOI:** 10.1093/bioadv/vbac010

**Published:** 2022-02-18

**Authors:** David Couvin, Alexis Dereeper, Damien F Meyer, Christophe Noroy, Stanie Gaete, Bernard Bhakkan, Nausicaa Poullet, Sarra Gaspard, Etienne Bezault, Isabel Marcelino, Ludovic Pruneau, Wilfried Segretier, Erick Stattner, Damien Cazenave, Maëlle Garnier, Matthieu Pot, Benoît Tressières, Jacqueline Deloumeaux, Sébastien Breurec, Séverine Ferdinand, Silvina Gonzalez-Rizzo, Yann Reynaud

**Affiliations:** 1 Unité Transmission, Réservoir et Diversité des Pathogènes, Institut Pasteur de Guadeloupe, Les Abymes, Guadeloupe 97139, France; 2 CIRAD, UMR ASTRE, Petit-Bourg, Guadeloupe 97170, France; 3 ASTRE, Univ Montpellier, CIRAD, INRAE, Montpellier 34000, France; 4 Développement, Analyse, Transfert et Application (DATA), Lamentin, Guadeloupe 97129, France; 5 Karubiotec Centre de Ressources Biologiques-UF 0216, CHU de la Guadeloupe, Pointe-à-Pitre 97110, France; 6 Registre des cancers de Guadeloupe, CHU de la Guadeloupe, Pointe-à-Pitre 97110, France; 7 URZ Recherches Zootechniques, INRAE, Petit-Bourg, Guadeloupe 97170, France; 8 Laboratoire COVACHIMM2E EA3592, Université des Antilles, Pointe-à-Pitre, Guadeloupe 97110, France; 9 UMR BOREA (MNHN, CNRS-7208, IRD-207, Sorbonne Université, UCN, UA), Université des Antilles, Pointe-à-Pitre, Guadeloupe 97110, France; 10 Équipe « Biologie de la mangrove » UMR7205 « ISYEB » MNHN-CNRS-Sorbonne Université-EPHE-UA, UFR SEN Département de Biologie, Université des Antilles, Pointe-à-Pitre, Guadeloupe 97110, France; 11 Laboratoire de Mathématiques Informatique et Applications (LAMIA), Université des Antilles, Pointe-à-Pitre, Guadeloupe 97110, France; 12 Centre d’Investigation Clinique Antilles Guyane, Inserm CIC 1424, Les Abymes, Pointe-à-Pitre, Guadeloupe 97110, France; 13 Faculté de Médecine Hyacinthe Bastaraud, Université des Antilles, Pointe-à-Pitre, Guadeloupe 97110, France

## Abstract

**Summary:**

Sequencing and other biological data are now more frequently available and at a lower price. Mutual tools and strategies are needed to analyze the huge amount of heterogeneous data generated by several research teams and devices. Bioinformatics represents a growing field in the scientific community globally. This multidisciplinary field provides a great amount of tools and methods that can be used to conduct scientific studies in a more strategic way. Coordinated actions and collaborations are needed to find more innovative and accurate methods for a better understanding of real-life data. A wide variety of organizations are contributing to KaruBioNet in Guadeloupe (French West Indies), a Caribbean archipelago. The purpose of this group is to foster collaboration and mutual aid among people from different disciplines using a ‘one health’ approach, for a better comprehension and surveillance of humans, plants or animals’ health and diseases. The KaruBioNet network particularly aims to help researchers in their studies related to ‘omics’ data, but also more general aspects concerning biological data analysis. This transdisciplinary network is a platform for discussion, sharing, training and support between scientists interested in bioinformatics and related fields. Starting from a little archipelago in the Caribbean, we envision to facilitate exchange between other Caribbean partners in the future, knowing that the Caribbean is a region with non-negligible biodiversity which should be preserved and protected. Joining forces with other Caribbean countries or territories would strengthen scientific collaborative impact in the region. Information related to this network can be found at: http://www.pasteur-guadeloupe.fr/karubionet.html. Furthermore, a dedicated ‘Galaxy KaruBioNet’ platform is available at: http://calamar.univ-ag.fr/c3i/galaxy_karubionet.html.

**Availability and implementation** Information about KaruBioNet is availabe at: http://www.pasteur-guadeloupe.fr/karubionet.html

**Contact:**

dcouvin@pasteur-guadeloupe.fr

**Supplementary information:**

Supplementary data are available at *Bioinformatics Advances* online.

## 1 Introduction

Rapid advances in sequencing technologies over the past quarter-century have led to substantial reductions in the cost of genome sequencing, producing huge amounts of heterogeneous data. Sequencing and biological associated data become more affordable and available. However, the information generated is difficult to analyze. Then, new tools and methods are therefore needed. 

As a French overseas territory, Guadeloupe is part of the European Union (EU) and belongs to its outermost regions. This archipelago is a small territory with an important local presence of national research organizations dealing with sequencing data. Bioinformatics is a multidisciplinary field that provides a large number of tools and methods that can be used to conduct scientific studies in a more strategic way. Until now, in Guadeloupe, this recent discipline was structured and coordinated at laboratory scale. In fact, most scientists used to favor the help of colleagues located outside the region and often long associated with the parent organization (mostly in mainland France) to perform the sequencing and analysis of their data. Although we can approve this practice for various needs of collaborations and strengthening of different projects, we believe that it would be beneficial for actors in the region to be able to access various resources and support platforms in bioinformatics and data sharing. Resources and networking are of primary interest to better establish collaboration between scientists (Hazbón *et al.*, 2018).

In the Caribbean, a bioinformatics and biostatistics network coordinating actions and collaborations was needed to find more innovative and accurate methods. As Karukera (‘the island of beautiful waters’) is the Native American name of Guadeloupe, we decided to create in January 2019 the Karukera Bioinformatics Network: KaruBioNet. However, we cannot omit lessons learnt from other renowned networks before establishing KaruBioNet such as EMBnet and H3ABioNet (D’Elia *et al.*, 2009; Mulder *et al.*, 2016).

Several organizations contribute to KaruBioNet. This network includes scientists from the Institut Pasteur de la Guadeloupe, the Université des Antilles (UA), the Centre de coopération internationale en recherche agronomique pour le développement (CIRAD), the Institut national de recherche pour l’agriculture, l’alimentation et l’environnement (INRAE), the Centre d’Investigation Clinique Antilles Guyane (CIC), the Institut national de la santé et de la recherche médicale (Inserm), the KarubiotecTM (Biological resources Center of University Hospital of Guadeloupe) and the Région Guadeloupe. The purpose of this group is to promote collaboration and mutual aid between people from different disciplines (biology, computer science, health informatics, biostatistics, mathematics, chemistry, epidemiology, ecology, clinical research, etc…). This transdisciplinary network is a platform for discussion, sharing, training and support between researchers, engineers and students interested in bioinformatics and related fields. Here, we aimed to establish and highlight the contribution of KaruBioNet to build solidarity links that allow better collaboration between researchers from various fields in Guadeloupe. Information related to this network can be found at: http://www.pasteur-guadeloupe.fr/karubionet.html. Furthermore, a dedicated ‘Galaxy KaruBioNet’ platform allowing to facilitate bioinformatic analyses is available at: http://calamar.univ-ag.fr/c3i/galaxy_karubionet.html. We also benefit from the ‘Exocet’ High-Performance Computing (HPC) facility of the UA to perform computing calculations.

## 2 Themes, tools and developments of a transdisciplinary network

### 2.1 Major common themes shared by laboratories

**Fig. 1.**
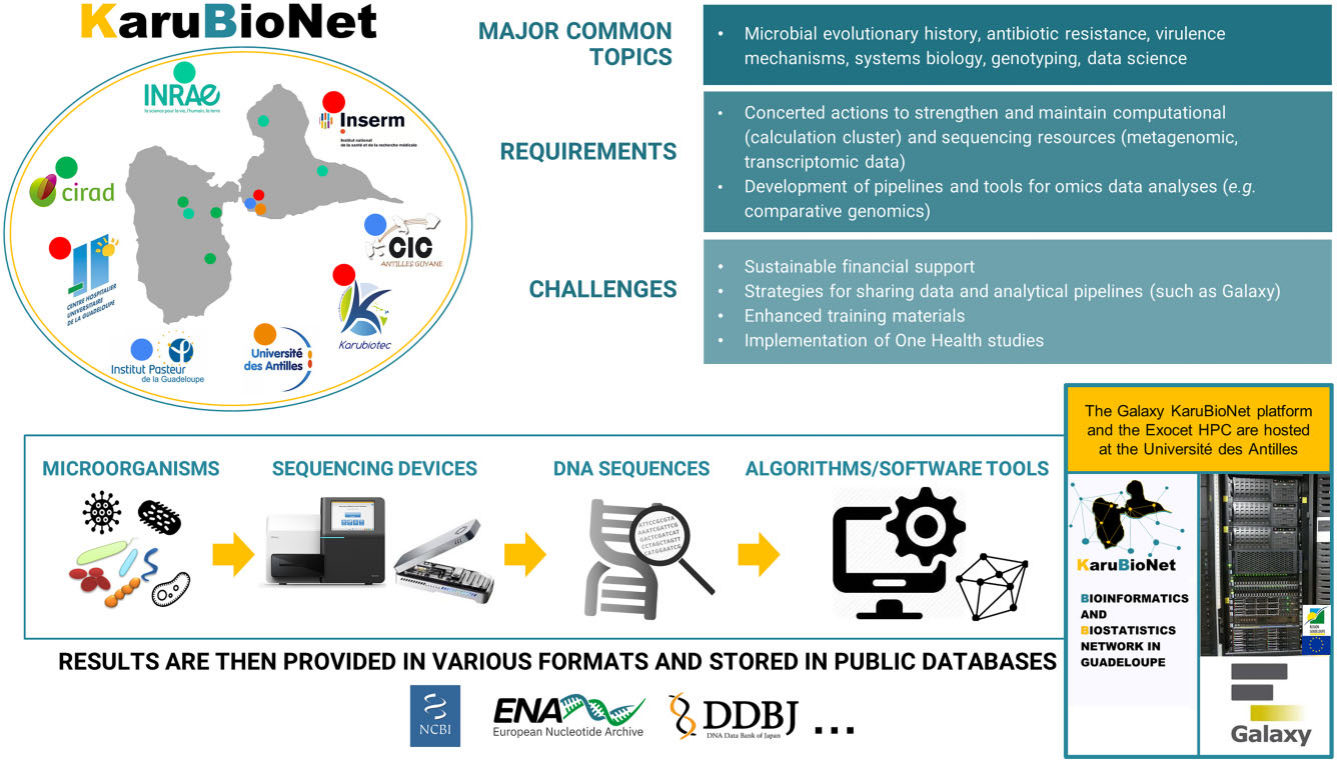
Mapping showing major common themes shared by research institutions involved in the KaruBioNet. The colored dots represent the location of each institution on the map. This figure also shows a simplified workflow for sequencing data analysis. Resources and platforms are mainly located at the UA

The main purpose of this group is to bring together people who share common problems to better collaborate together. The KaruBioNet network aims to improve the development of bioinformatics at the local or regional level for a better understanding and analysis of real-life data. The major common themes shared by laboratories belonging to the network are microbial evolutionary history, antimicrobial resistance, virulence mechanisms, systems biology, genotyping and data science (Fig. 1). The KaruBioNet network particularly aims to help researchers in their studies related to metagenomics, proteomics, genomics (or other ‘omics’), as well as more general aspects relating to data analysis, integration and interpretation. In order to structure discussions and exchanges between researchers, we have implemented different topics (which could evolve in the future): (i) omics sciences; (ii) artificial intelligence and machine learning; (iii) biochemical analyses; (iv) geographic information systems; (v) databases and software development; (vi) biostatistics; and (vii) epidemiology. A video channel was created to disseminate training materials in French as well as presentations or demonstrations related to bioinformatics (details are provided in Supplementary Data).

### 2.2 Needs and challenges specific to omics sciences

Concerted actions are needed to strengthen and maintain computational and sequencing resources between institutions. We also intend to promote the development of dedicated bioinformatics pipelines and tools specific to omics data analysis. Several future challenges are foreseeable: (i) efforts will be necessary to find sustainable financial support to maintain the network; (ii) new and innovative strategies will be needed to share data and analytical pipelines (like Galaxy); and (iii) improved training materials will promote the development of bioinformatics in our region and elsewhere.

This would allow analytical strategies to be built around the One Health concept defined as a collaborative, multisectoral and transdisciplinary approach working at local, regional, national and global levels, to achieve optimal health outcomes and recognize the interconnection between people, animals, plants and their common environment (Lerner and Berg, 2017). This approach would make the challenge relevant for a better understanding and surveillance of human, plant or animal health and diseases (Gruel *et al.*, 2021).

## 3 Sequencing techniques

Sequencing devices are currently available in Guadeloupe. Devices such as Nanopore MinION (https://nanoporetech.com/products/minion) or Illumina MiSeq (https://emea.illumina.com/systems/sequencing-platforms/miseq.html) can be used for real-time sequencing projects.

Various sequencing projects are ongoing in several labs involved in the KaruBioNet. The impact of bacterial genomes such as *Klebsiella pneumoniae*, *Escherichia coli* or *Enterobacter cloacae* are being studied in depth in our environment (notably their ability to be resistant to some antibiotics; Pot *et al.*, 2021). Moreover, to study bacterial adaptations to environmental changes or the interaction with organisms in the same environment, RNA sequencing is a powerful tool. It makes it possible to study the differential expression of messenger RNAs and to identify potential regulators of non-coding RNAs. RNA sequencing generates a lot of data and requires the help of specific software accessible in the KaruBioNet network. A simplified workflow for sequencing data analysis is shown in Figure 1. A wide variety of heterogeneous sequencing data is produced and analyzed within the network (details are provided in Supplementary Data).

## 4 Exocet computing cluster

The ‘Exocet’ HPC facility of the UA provides us a great capacity for analyzing whole-genome sequencing (WGS) data. With almost 1000 CPU cores and 7349 GB RAM, Exocet cluster contains powerful nodes and NVidia graphics cards allowing it to perform bioinformatics analyses for all members of the KaruBioNet team.

Resources are shared among all members of KaruBioNet via access to Exocet HPC. Users must make a request to the Center Commun de Calcul Intensif of the UA (http://calamar.univ-ag.fr/c3i/exocet.html). All members of the network can access Exocet and other services free of charge. KaruBioNet administrators manage dedicated servers and other services. We promote our various services where possible to facilitate their use.

Exocet contains:

- Two front-end servers named ‘exocet1’ and ‘exocet2’. When we log into Exocet, we are actually logging into one of the two servers.
- Twenty-five ‘Calculation’ nodes (node01 to node25) are used for general calculations on Intel processors. Each node contains two Intel processors of 18 cores each, making a total of 36 cores for one node, and 192 GB of RAM.
- A ‘large RAM’ node (mem01) with the same two Intel processors as a ‘Calculation’ node but with 1536 GB of RAM memory.
- A ‘V100’ node (gpu01) having the same characteristics as a ‘Calculation’ node but with in addition two Nvidia TESLA V100 graphics cards.
- A ‘T4’ node (gpu02) similar to the ‘V100’ but with two NVidia T4 graphics cards.
- Dual-processor servers each with 256 GB of RAM memory and a QUADRO P5000 graphics card.

The mobaXterm application (https://mobaxterm.mobatek.net/) can be used to get connected to the computing cluster.

## 5 Galaxy KaruBioNet platform

We have started the installation of a local Galaxy platform (http://galaxyproject.org/; Afgan *et al.*, 2018) that will allow researchers and students who are not comfortable with command-line interfaces to perform bioinformatic analyzes in a user-friendly manner. This local platform is available online from a calculation server of the UA. Users need to register to the Galaxy platform using a login (email address) and a password (http://calamar.univ-ag.fr/c3i/galaxy_karubionet.html). This local Galaxy instance (hosted at the UA) can be considered as a showcase reflecting different themes and activities of the network (Fig. 1).

### 5.1 Dedicated specialized workflows

#### 5.1.1 Bacterial pangenomics

**Fig. 2.**
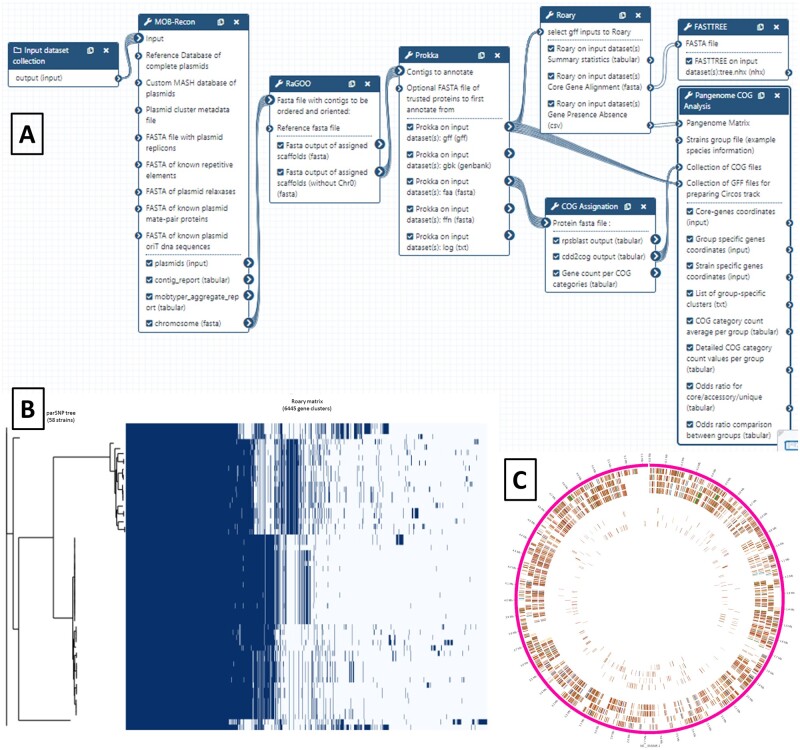
Galaxy workflow and its graphical outputs for pangenome analysis of bacterial strains. (**A**) Bacterial pangenomics Galaxy workflow. (**B**) Gene presence/absence matrix facing the phylogenetic tree based on core-genome alignment. (**C**) Circos representation of core genes and specific genes

A ready-to-use workflow is available for Galaxy KaruBioNet users to process a complete analysis of the bacterial pangenome/core-genome. Starting from a collection of strains for which WGS reads are available, the user will be able to process successively analytical steps from raw reads to fully assembled and annotated genomes, and then a comparative genomics analysis to finally obtain both a pangenome matrix image and a Circos (Krzywinski *et al.* 2009) representation of pangenome ready for publication (Fig. 2). The first part of the workflow takes advantage of the Galaxy dataset collections to reconstruct and annotate complete individual genomes for each strain (each program will be executed as many times as there are strains to analyze):

1. *De novo* assembly of Illumina reads with Unicycler (Wick *et al.*, 2017).
2. Assignation of contigs to plasmids or chromosomal with MOB-Recon (Robertson and Nash, 2018).
3. Anchoring and ordering of scaffolds using RaGOO (Alonge *et al.*, 2019), in order to establish a complete pseudomolecule for the chromosomal genome. This step requires an external reference genome.
4. Structural and functional annotation of reconstructed genomes using Prokka (Seemann, 2014).
5. COG (Cluster of Orthologs Groups; Galperin *et al.*, 2021) assignation of genes.

The second part of the workflow process is linear (each program will be executed only once) and allows the comparison of genomes:

1. Pangenome analysis using Roary (Page *et al.*, 2015).
2. Pangenome matrix representation using roary_plots.
3. Core-genome-based phylogeny using FastTree (Price *et al.*, 2010).
4. Circular representation of core genes and strain-specific genes positioned on a reference genome, using Circos.

#### 5.1.2 *Mycobacterium tuberculosis* genomic analyses

Tuberculosis (TB) is an infectious disease caused by bacteria belonging to the *Mycobacterium tuberculosis* complex (MTBC). Interestingly, several Galaxy tools exist to study MTBC strains from available sequence reads or assembled genomes. Resources related to *M.tuberculosis* were tentatively listed in the MTBCtools list (Couvin *et al.*, 2021) in which tools were classified into several thematic sections/categories such as biological databases, drug resistance or prediction/classification tools. The Galaxy ToolShed (https://toolshed.g2.bx.psu.edu/) contains several TB tools such as Galru (Page *et al.*, 2020), lorikeet spoligotyping (Cohen *et al.*, 2015), Mykrobe (Hunt *et al.*, 2019), SpoTyping (Xia *et al.*, 2016) and TB-Profiler (Phelan *et al.*, 2019). We have recently added MIRUReader (Tang and Ong, 2020) to the Galaxy ToolShed. These tools could be used to produce genotyping information such as spoligotypes (https://fr.wiktionary.org/wiki/spoligotype) or Mycobacterial interspersed repetitive units-variable number of tandem DNA repeats (MIRU-VNTRs), and detect drug resistance profiles from MTBC DNA sequences.

An example of a workflow to study the genotyping of *M.tuberculosis* strains and their association with antibiotic resistance would be as follows:

(i) users can analyze their sequence reads using the lorikeet spoligotyping program to obtain spoligotypes matching against their reads. Furthermore, they can use TB-Profiler program to determine the drug resistance profile from reads; (ii) they can then use a *denovo* assembly program such as Shovill (https://github.com/tseemann/shovill), SPAdes (Bankevich *et al.*, 2012) or Unicycler; and (iii) MIRUReader tool can finally be used to determine 24-loci MIRU-VNTRs profiles from preassembled data.

### 5.2 Other examples of modules/workflows

#### 5.2.1 Simple phylogeny workflow

Phylogenetic analyzes are widely used to study biological data and other scientific data. From a Multi-FASTA file, users can perform a multiple alignment using a multiple alignment program such as MAFFT (Katoh and Standley, 2013). Users can then perform a phylogenetic analysis with a dedicated program such as FastTree, PhyML (Guindon *et al.*, 2010) or RAxML (Stamatakis, 2014). Note that user-friendly approaches such as Phylogeny.fr or NGPhylogeny.fr (Dereeper *et al.*, 2008; Lemoine *et al.*, 2019) already exist to perform phylogenetic analyses. The phylogenetic trees obtained can be annotated using iTOL (Letunic and Bork, 2021).

#### 5.2.2 Construction of a variant call format file and single nucleotide polymorphism analyses

To construct a variant call format file, users can choose the BWA-MEM (Li, 2014) tool to map their sequence reads against a reference genome, then they can use Samtools/mpileup to perform a multi-way pileup of variants (Li *et al.*, 2009). Finally, they can use the VarScan (Koboldt *et al.*, 2012) program for variant detection. Other Galaxy pipelines such as Snippy (https://github.com/tseemann/snippy) could be used for single nucleotide polymorphism analyses.

#### 5.2.3 Automated pipeline for the search for resistance, plasmid and virulence genes

We have developed a pipeline called catchSequenceInfo that allows us to get resistance, virulence and plasmids, as well as multilocus sequence typing (MLST; Jolley *et al.*, 2018) information from DNA sequences. This tool uses: (i) ABRicate (https://github.com/tseemann/abricate) with ResFinder (Zankari *et al.*, 2012), PlasmidFinder (Carattoli *et al.*, 2014) and VFDB (Liu *et al.*, 2019) databases to predict resistance, plasmid and virulence genes; as well as (ii) MLST (https://github.com/tseemann/mlst) to get allele IDs and MLST number. catchSequenceInfo is freely available through the Galaxy KaruBioNet instance and it has been placed in the Galaxy ToolShed. Note that the pMLST tool (Carattoli *et al.*, 2014) has also been installed in our Galaxy instance as well as in the ToolShed.

### 5.3 A local galaxy training focused on metagenomics

Metagenomic analyses supported by high-throughput sequencing provide a method to evaluate the microbial community in terms of both taxonomy and potential functioning.

A local Galaxy training took place on June 17, 2021 at the UA. During this training session, we used the Galaxy Training webpage (https://training.galaxyproject.org/) to practice with a tutorial titled Galaxy 101 (https://training.galaxyproject.org/training-material/topics/introduction/tutorials/galaxy-intro-101/tutorial.html). Then, we used another tutorial to perform metabarcoding analyses using the FROGS pipeline (Escudié *et al.*, 2018). This pipeline has been shown to be very effective for metagenomics studies. It could also be coupled with the analysis pipeline used in SHAMAN (Volant *et al.*, 2020).

## 6 Databases and data warehouses

Some developments are ongoing to construct dedicated databases or data warehouses.

The SITVIT databases and other resources (Couvin *et al.*, 2017, 2022, 2019, 2020; Demay *et al.*, 2012) developed by researchers at the Institut Pasteur de la Guadeloupe have provided a more complete global view of the molecular epidemiology of MTBC for public health surveillance. These resources are available online and may be of use to researchers working specifically on these bacterial organisms.

Bacterial pathogens have evolved specific effector proteins to exploit host cell machinery and hijack the immune responses during infection. Dedicated multiprotein complexes, known as secretion systems, secrete these effectors. Type IV secretion systems (T4SS) are specialized adenosine triphosphate-dependent protein complexes used by many bacterial pathogens for the delivery of Type IV Effectors (T4Es) proteins into eukaryotic cells to subvert host cell processes during infection. To help biologists to identify putative T4Es from bacterial genomes, S4TE2.0 (Meyer *et al.*, 2013; Noroy *et al.*, 2019; Noroy and Meyer, 2021), an online bioinformatic suite of tools has been developed by researchers from KaruBioNet at CIRAD Guadeloupe. S4TE 2.0 predicts and ranks candidate T4Es by using a combination of 14 sequence characteristics, including homology to known effectors, homology to eukaryotic domains, presence of subcellular localization signals or secretion signals, etc. S4TE 2.0 generates a score and sorts the best T4Es candidates. S4TE-CG allows the comparison of up to four predicted type IV effectomes.

The following table shows various tools and databases which are available for studying specific aspects of infectious diseases (such as *M.tuberculosis* genotyping information or Type IV effector proteins; Table 1).

**Table 1.** Selected databases and resources available in the network

Name	Description	Link
SITVITWEB	MTBC genotyping database containing information on 62 582 isolates	http://www.pasteur-guadeloupe.fr:8081/SITVIT_ONLINE/
SITVIT2	An update of SITVITWEB containing additional molecular markers and information on 111 635 isolates	http://www.pasteur-guadeloupe.fr:8081/SITVIT2
SITVITBovis	A publicly available database and mapping tool to get an improved overview of animal and human cases caused by *Mycobacterium bovis* (*n* = 25 741 isolates)	http://www.pasteur-guadeloupe.fr:8081/SITVIT_Bovis
SpolSimilaritySearch	Similarity search algorithm of spoligotype patterns in the SITVIT2 database	http://www.pasteur-guadeloupe.fr:8081/SpolSimilaritySearch/
SpolLineages	A tool to predict MTBC families from spoligotyping or MIRU-VNTR patterns	http://www.pasteur-guadeloupe.fr:8081/SpolLineages/
S4TE2.0	A tool to predict T4SS effectors from bacterial genomes	https://sate.cirad.fr/S4TE.php
S4TE-EM	A tool based on the S4TE 2.0 algorithm which allows users to adjust all parameters	https://sate.cirad.fr/S4TE-EM.php
S4TE-CG	A tool to compare up to four effectomes predicted by SATE 2.0 algorithm	https://sate.cirad.fr/S4TE-CG.php

The links of all these databases developed inside KaruBioNet are available on the website. The development of collaborations will lead to the creation of new databases or to the use of existing databases. The sensitivity of these data (personal data, health data, genetic data, etc.) requires compliance with European data protection rules (GDPR).

## 7 Machine learning and classification problems

**Fig. 3.**
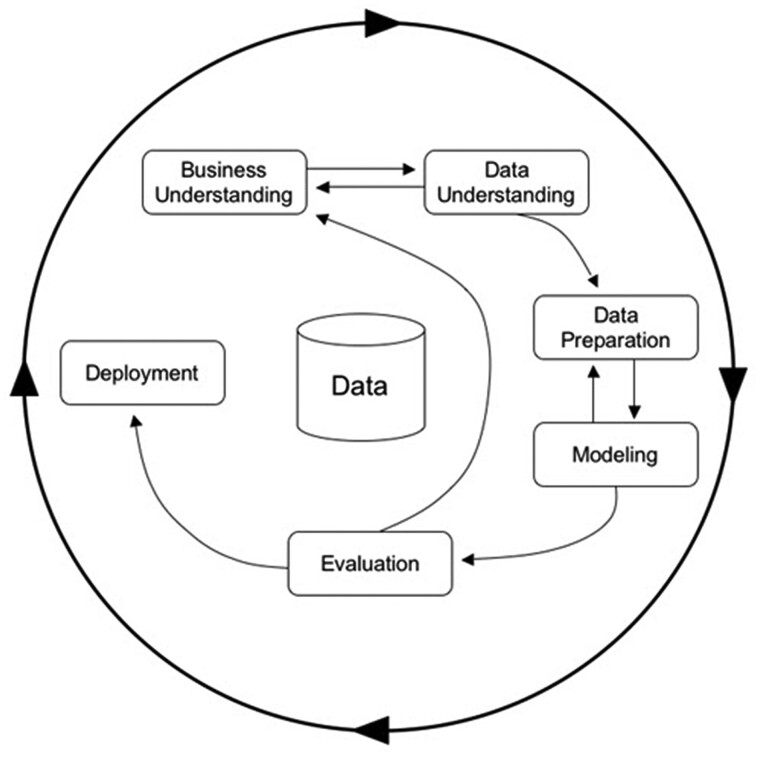
Cross Industry Standard Process for Data Mining (Crisp-DM) model that describes common approaches used by KDD and data mining experts

Knowledge Discovery from Databases (KDD) is the ‘non-trivial process of identifying valid, novel, potentially useful, and ultimately understandable patterns in data’ (Fayyad *et al.*, 1996). Over the last 30 years, advanced prediction methods and tools borrowed to the KDD field, have contributed to defining new kinds of prediction models, namely data-driven models. They consist of looking for correlations between predictive historical variables and output variables. When the output variables are discrete, the problems considered are referred to as ‘classification problems’. Figure 3 presents the different steps of a KDD approach. First, it is important to understand the data and the associated context, then a preparation step (also known as preprocessing) is often necessary in order to overcome problems such as missing data, normalization issues, noise reduction or data transformation, and obtain usable data to feed a dedicated model. Among the current models used to tackle these problems, artificial neural networks (ANNs), decision trees or support vector machines are the most common. They have proved to be very effective compared to knowledge-driven solutions with the advantage of requiring less domain knowledge for their design. However, one of the issues in data-driven approaches is the understandability and readability of models that end-users should trust. Indeed, a lot of techniques, including ANNs, can be seen as delivering black-boxes since they do not provide explanations of how they work. Decision-makers are more likely to trust models whose predictions are interpretable and understandable.

## 8 Conclusion and perspectives

Through this network, we aim to forge links of solidarity allowing a better collaboration between researchers from various fields. Concerted, collaborative and coordinated actions are still needed to better structure bioinformatics in our environment. Indeed, several examples of well-established bigger structures at national level already exist such as the French Institute of Bioinformatics (https://www.france-bioinformatique.fr/en), the Center of Bioinformatics Biostatistics and Integrative Biology (https://c3bi.pasteur.fr/) or the South Green Bioinformatics platform (https://www.southgreen.fr/; South Green Collaborators, 2016). Other examples of structuring are visible at a wider scale. Furthermore, educational initiatives such as Meet-U (http://www.meet-u.org/) are inspiring to set up innovative and original learning processes and collaboration (Abdollahi *et al.*, 2018). Starting from a little archipelago in the Caribbean, we envision to facilitate exchange with other Caribbean partners in the future, knowing that the Caribbean is a region with a non-negligible biodiversity, which should be preserved and protected. Joining forces with other ‘isolated’ Caribbean countries or territories would strengthen scientific collaborative impact in the region (Degrave *et al.*, 2002; Gaete and Deloumeaux, 2017). Finally, the KaruBioNet network could potentially evolve by collaborating with other Caribbean countries, and give rise to another greater network tentatively called KariBioNet. Further efforts are needed to achieve this goal. However, the actions initiated by the launch of an open-access Galaxy instance and a website bringing together our activities could help many users in the analysis of their data.

## Supplementary Material

vbac010_Supplementary_Data
