# Supplementary material for: KaruBioNet: a network and discussion group for a better collaboration and structuring of bioinformatics in Guadeloupe (French West Indies)
Source: Bioinform Adv. 2022 Feb 18;2(1):vbac010. doi: 10.1093/bioadv/vbac010 (PMC9710593; doi:10.1093/bioadv/vbac010)
Supplement: vbac010_Supplementary_Data [file vbac010_supplementary_data.pdf]

Supplementary Data

KaruBioNet: a network and discussion group for a better collaboration and structuring of bioinformatics in Guadeloupe (French West Indies)

David Couvin, Alexis Dereeper, Damien F. Meyer, Christophe Noroy, Stanie Gaete, Bernard Bhakkan, Nausicaa Poullet, Sarra Gaspard, Etienne Bezault, Isabel Marcelino, Ludovic Pruneau, Wilfried Segretier, Erick Stattner, Damien Cazenave, Maëlle Garnier, Matthieu Pot, Benoît Tressières, Jacqueline Deloumeaux, Sébastien Breurec, Séverine Ferdinand, Silvina Gonzalez-Rizzo, Yann Reynaud, for the KaruBioNet team

Various dissemination methods

<u>A dedicated website</u>

The development of a dedicated website (Supplementary Figure S1) has also been started to facilitate the connection between the involved actors and various activities. Waiting for a better Web server environment, it is temporarily available at the following link: http://www.pasteur-guadeloupe.fr/karubionet.html. Furthermore, key information could also be consulted on a dedicated Google Drive.

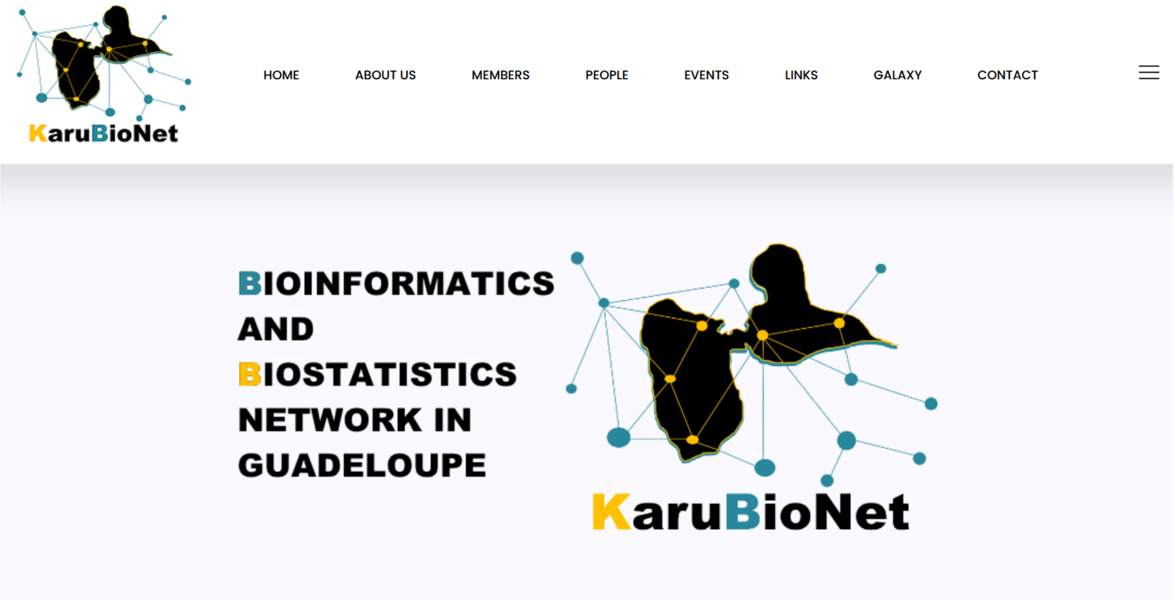

Supplementary Figure S1: Snapshot of the dedicated website under construction.

<u>Karubiotools GitHub page</u>

A dedicated GitHub page (https://github.com/karubiotools) allows us to deposit computer codes/repositories developed by our team or by others (forked repositories), and it facilitates reproducibility of codes and their sharing. This page also provides a way to keep code history and provide traceability. Findable, Accessible, Interoperable, Reusable (FAIR) methods could also foster reproducibility of codes and help in the development of novel specific tools. Other documents and training materials can be placed on this GitHub page.

YouTube channel
A dedicated YouTube channel named "KaruBioNet Channel" (https://www.youtube.com/channel/UCrX_kfFGF7mMR-_-wiv5O6Q) has been set up to disseminate the presentations/demonstrations and training carried out through the network.

Perspectives on educational activities
In addition, several actions are set up in order to better include bioinformatics in courses offered by the Université des Antilles and to popularize this (re-)emerging scientific field. Some bioinformatics teaching modules ("EC libres") are planned within the Université des Antilles to improve the development of the field locally. The Galaxy KaruBioNet local instance can also be used to develop teaching programs or dedicated training.

Sharing of existing tools and tools to be developed
A wide range of bioinformatic tools have been added to the Galaxy KaruBioNet. Other tools will be added as we go.

**Existing tools**	**Tools to be added**
ABRicate, MOB-Recon	RFPlasmid, PlasFlow, mlplasmids, …
Samtools	ResFinder
MEGAHIT, Shovill, SPAdes, Unicycler, …	CRISPRCasFinder
FASTQc, MultiQC, QUAST	CGview, EasyFig
Snippy, SNiPlay, ...	MinIONQC
MLST, PMLST, Kleborate, Prokka, …	…
MAFFT, MUSCLE, PhyML, RAxML, ...	
MIRUReader, Mykrobe, TB-Profiler, …	
FROGS	

A more complete list will be added to the Google Drive of the KaruBioNet team. Furthermore, an Excel file containing further details regarding themes and topics shared by members of the network, will be available soon.
